# Asymptotic Capacity Results on the Discrete-Time Poisson Channel and the Noiseless Binary Channel with Detector Dead Time

**DOI:** 10.3390/e22080846

**Published:** 2020-07-30

**Authors:** Ligong Wang

**Affiliations:** ETIS Laboratory, UMR 8051—CY University, ENSEA, CNRS, 95014 Cergy, France; ligong.wang@ensea.fr

**Keywords:** channel capacity, channels with memory, dead time, Poisson channel, wideband

## Abstract

This paper studies the discrete-time Poisson channel and the noiseless binary channel where, after recording a 1, the channel output is stuck at 0 for a certain period; this period is called the “dead time.” The communication capacities of these channels are analyzed, with main focus on the regime where the allowed average input power is close to zero, either because the bandwidth is large, or because the available continuous-time input power is low.

## 1. Introduction

**Figure 1:**
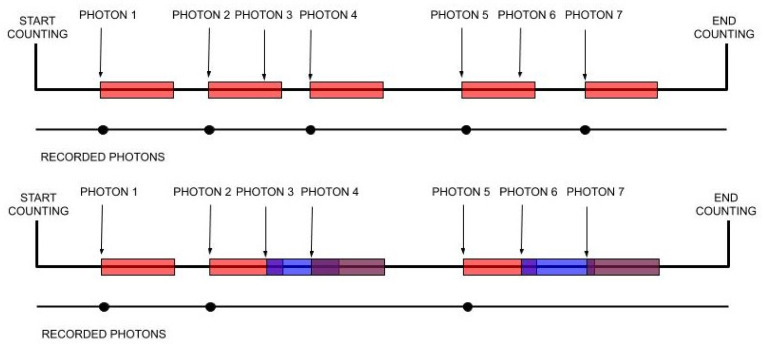
Nonparalyzable (**top**) and paralyzable (**bottom**) dead time. The colored rectangles indicate the dead-time periods.

For some detection systems that record arrivals, for example, a single-photon avalanche diode, after each recorded arrival, there comes a period during which the detector is not able to record any new arrivals. This period is often called the “dead time”; see, e.g., [1,2,3]. There are mainly two types of behavior for the dead time: nonparalyzable (nonextendable) means the detector dead time is a fixed period after each recorded arrival; and paralyzable (extendable) means the dead time happens after each occurred arrival, i.e., an arrival that is not recorded because the channel is already “dead” will restart the dead-time period. The two types of dead time are illustrated in Figure 1.

One important example of a communication system where the detector may be affected by dead time is direct-detection optical communication, where the input signal is emitted by a laser or light-emitting diode. In Information Theory, such a system is often modeled as a Poisson channel. Some capacity results on the Poisson channel can be found in [4,5,6,7,8,9,10,11]. In this work we are interested in the communication capacity of the discrete-time Poisson channel with detector dead time, which, to our knowledge, is largely unexplored.

Before attacking the Poisson channel, we first look at the simpler problem of the noiseless binary channel, where the input can be 0 or 1, and where, when the channel is not “dead,” the channel output always equals the input. We think of each received 1 as an arrival, and assume that it triggers a dead-time period of *d* channel uses. This problem has been extensively studied in the literature on run-length limited (RLL) coding [12,13,14,15]; we shall elaborate on this later. The noiseless binary channel with dead time can be thought of as a model for direct-detection optical channel with number states containing zero or one photon as inputs.^1^ Furthermore, as we shall see, it serves as a reference for comparison for the Poisson channel.

For both channels, we are primarily interested in the wideband regime, where each channel use corresponds to a short time duration. We believe this regime to be particularly relevant, because each dead-time period would occupy a large number of channel uses. When studying the Poisson channel in the wideband regime, we distinguish between the cases where there is feedback and where there is not. Related to wideband, we also study the low-continuous-time-power regime where bandwidth is moderate. In both regimes, the average input power per channel use is small, but in the low-continuous-time-power regime, the dead-time period only occupies a moderate number of channel uses.

For most of the above cases, we determine the asymptotic capacity; in some cases we also determine the second-order term. In these cases, we show that dead time does not affect the asymptotic capacity. An exception is the Poisson channel in the wideband regime without feedback, for which we prove a lower bound, but we have not found a matching upper bound. In this case, we suspect that dead time does incur a penalty on the dominant term in capacity.

After introducing some notation and definitions, we present first our study on the noiseless binary channel, and then that on the Poisson channel. At the end of the paper, we summarize the results and discuss some future research directions.

### Some Notation and Definitions

The channel, whether it is noiseless or Poisson, is characterized by two parameters: β denotes the maximum allowed average input power per channel use, and *d* denotes the duration of each dead-time period in channel uses. We further denote

(1) η≜dβ,

which corresponds to the maximum allowed average input energy per dead-time period.

Capacity is in discrete time and has the unit “nats per channel use.” As usual, it is defined as the supremum over all communication rates for which the probability of a decoding error can be made arbitrarily small. Given the above two parameters β and *d*, we use $C^{\mathrm{NL}}\left(\beta ,d\right)$ to denote the capacity of the noiseless binary channel, $C^{P}\left(\beta ,d\right)$ that of the Poisson channel without feedback, and $C_{\mathrm{FB}}^{P}\left(\beta ,d\right)$ that of the Poisson channel with feedback. Sometimes, to highlight the fact that we hold the parameter η to be fixed, we write $\frac{\eta }{\beta }$ in place of *d*, such as in $C^{P}\left(\beta ,\frac{\eta }{\beta }\right)$.

Unless otherwise stated, we use O(·) and o(·) to describe functions of β in the regime where β tends to zero, hence a function described as O(f) satisfies

(2) $$\underset{\beta ↓0}{lim sup}\left|\frac{O(f)}{f}\right|<\infty ,$$

and a function described as o(f) satisfies

(3) $$\lim_{\beta ↓0}\frac{o(f)}{f}=0.$$

Sometimes the dependence of the function *f* on β may be implicit, and *f* may be the constant 1.

Throughout this paper, log denotes the natural logarithm, and information is measured in nats.

## 2. The Noiseless Binary Channel

We consider a noiseless channel with dead time *d*, where the input *X* takes value in {0,1}. The law of the channel for nonparalyzable dead time is described as

(4) $$\begin{array}{ccc} Pr(Y_{i}=1|X^{i}=x^{i},Y^{i-1}=y^{i-1}) & = & \left\{\begin{array}{cc} 1, & \mathrm{if}\;x_{i}=1\;\mathrm{and}\;y_{j}=0\;\mathrm{for}\;\mathrm{all}\;j\in \left\{i-d,\ldots ,i-1\right\}\\ 0, & \mathrm{otherwise}. \end{array}\right. \end{array}$$

For paralyzable dead time, the channel law is given by

(5) $$\begin{array}{ccc} Pr(Y_{i}=1|X^{i}=x^{i},Y^{i-1}=y^{i-1}) & = & \left\{\begin{array}{cc} 1, & \mathrm{if}\;x_{i}=1\;\mathrm{and}\;x_{j}=0\;\mathrm{for}\;\mathrm{all}\;j\in \left\{i-d,\ldots ,i-1\right\}\\ 0, & \mathrm{otherwise}. \end{array}\right. \end{array}$$

Without constraints on the input sequence, the capacity of the channels (4) and (5) is well understood in terms of RLL codes. For example, it is given by the logarithm of the largest root to [13]

(6) $$a^{d+1}-a^{d}-1.$$

Alternatively, it can be written as

(7) $$\begin{array}{c} \max_{\alpha \in [0,1]}\frac{1}{1+d\alpha }H_{b}\left(\alpha \right), \end{array}$$

where $H_{b}\left(\cdot \right)$ denotes the binary entropy function:

(8) $$\begin{array}{c} H_{b}\left(a\right)≜a\log\frac{1}{a}+\left(1-a\right)\log\frac{1}{1-a},\;a\in \left[0,1\right]. \end{array}$$

Now consider the case where an average-power constraint is imposed on the input sequence. Specifically, for a codebook at blocklength *n*, we require that the average number of 1s contained in a codeword not exceed nβ. The capacity of this power-constrained channel is again an elementary result. For completeness, we present it as a proposition and provide a proof.

**Proposition** **1.** *The capacities of the noiseless binary channels (4) and (5) with dead time d subject to the above power constraint are equal, and are given by*

(9) $$\begin{array}{ccc} C^{NL}\left(\beta ,d\right) & = & \max_{\alpha :\frac{\alpha }{1+d\alpha }\leq \beta }\frac{1}{1+d\alpha }H_{b}\left(\alpha \right). \end{array}$$

**Proof.**  See Appendix A. □

Before proceeding with the asymptotic analysis, we put the above channel model in a continuous-time perspective. To this end, consider a noiseless photon channel where the receiver employs a single-photon detector with a time resolution of *t* seconds. More precisely, the time axis is divided into slots each lasting *t* seconds, and the detector declares each detected photon as belonging to a certain slot; it is not able to provide finer timing information on the photons. The sender sends a sequence of photons into the channel, each at a chosen time.^2^ We impose an input-power constraint which says that the transmitter can send at most ρ photons per second. Assume that each dead-time period lasts τ seconds, where τ is an integer multiple of *t*. We can now think of each *t*-second slot as one use of a discrete-time noiseless binary channel. The discrete-time input is 1 if and only if the sender sends at least one photon in this slot. (Note that the transmitter’s choice of timing within that slot is irrelevant, because the receiver cannot recover this information. Note as well that it is suboptimal for the transmitter to send more than one photon within a slot, because the second photon cannot be detected.) The discrete-time output is 1 if and only if a photon is detected in this slot. The discrete-time input-power constraint is β=ρt, and the discrete-time dead-time duration is $d=\frac{\tau }{t}$ channel uses.

In the following, we study the wideband regime where *t* is brought down to zero while ρ is held fixed. In this case, β approaches zero proportionally to *t*, while *d* grows to infinity proportionally to $\frac{1}{t}$; the product η=dβ=τρ remains unchanged. Then we also study the low-continuous-time-power regime where ρ is brought down to zero while *t* is held fixed. In this case, *d* is fixed and finite when β↓0. For clarity, in the rest of this section we shall stay with the discrete-time picture, and only use the parameters *d*, β, and η.

As a reference for comparison, we recall that the capacity of the noiseless binary channel without dead time, in the regime where the average power β↓0, is given by $H_{b}\left(\beta \right)$ and behaves like

(10) $$\beta \log\frac{1}{\beta }+\beta +o\left(\beta \right).$$

We note that (10) has the same dominant term as the asymptotic capacity of the Poisson channel (without dead time), but a better second-order term [9,10]; see also (47) ahead.

### 2.1. Wideband Regime

We consider the regime where β↓0 while η=dβ is held fixed. As we shall see, the asymptotic capacity has different expressions when η<1 and when η≥1.

#### 2.1.1. The Case η<1

In this case, the following proposition shows that the detector dead-time effect on $C^{NL}\left(\beta ,\frac{\eta }{\beta }\right)$ is on the second-order term.

**Proposition** **2.** *In the regime where β↓0 and η=dβ is fixed and less than 1,*

(11) $$\begin{array}{ccc} C^{NL}\left(\beta ,\frac{\eta }{\beta }\right) & = & \beta \log\frac{1}{\beta }+\beta +\beta \log\left(1-\eta \right)+o\left(\beta \right). \end{array}$$

**Proof.**  First, note that, when η<1, the condition for the maximization in (9) is equivalent to

(12) $$\begin{array}{ccc} \alpha & \leq & \frac{\beta }{1-d\beta }=\frac{\beta }{1-\eta }. \end{array}$$

When β is sufficiently small, one could verify that $\frac{1}{1+d\alpha }H_{b}\left(\alpha \right)$ is monotonically increasing in α satisfying (12). Consequently, (for sufficiently small β) the maximum in (9) is achieved by $\alpha =\frac{\beta }{1-\eta }$, and

(13) $$\begin{array}{ccc} C^{NL}\left(\beta ,\frac{\eta }{\beta }\right) & = & \left(1-\eta \right)\left(\frac{\beta }{1-\eta }\log\frac{1-\eta }{\beta }+\frac{1-\beta -\eta }{1-\eta }\log\frac{1-\eta }{1-\beta -\eta }\right) \end{array}$$

(14) $$\begin{array}{rr} = & \beta \log\frac{1}{\beta }+\left(1-\eta -\beta \right)\log\frac{1}{1-\beta -\eta }-\left(1-\eta \right)\log\frac{1}{1-\eta }. \end{array}$$

The expression (11) follows by applying Taylor series expansion and rearranging terms. □

#### 2.1.2. The Case η≥1

In this case, dead time incurs a penalty on the first-order term of $C^{NL}\left(\beta ,\frac{\eta }{\beta }\right)$ as shown by the following proposition.

**Proposition** **3.** *In the regime where β↓0 and η=dβ is fixed and greater than or equal to 1,*

(15) $$\begin{array}{c} C^{NL}\left(\beta ,\frac{\eta }{\beta }\right)=\frac{\beta }{\eta }\log\frac{1}{\beta }+o\left(\beta \log\frac{1}{\beta }\right). \end{array}$$

**Proof.**  We first note that the condition in (9) always holds. Indeed, when η≥1, we have

(16) $$\begin{array}{ccc} \frac{\alpha }{1+d\alpha } & < & \frac{\alpha }{d\alpha }=\frac{1}{d}\leq \frac{\eta }{d}=\beta . \end{array}$$

Hence the average-power constraint is inactive.

We now derive a lower bound on $C^{NL}\left(\beta ,d\right)$. To this end, we bound

(17) $$\begin{array}{ccc} C^{NL}\left(\beta ,d\right) & = & \max_{\alpha \in [0,1]}\frac{1}{1+d\alpha }\left(\alpha \log\frac{1}{\alpha }+\left(1-\alpha \right)\log\frac{1}{1-\alpha }\right) \end{array}$$

(18) $$\begin{array}{rr} \geq & \max_{\alpha \in [0,1]}\frac{\alpha }{1+d\alpha }\cdot \log\frac{1}{\alpha }. \end{array}$$

With the specific choice $\alpha =\frac{1}{d}\log d$, we obtain

(19) $$\begin{array}{ccc} C^{NL}\left(\beta ,d\right) & \geq & \frac{1}{1+\log d}\cdot \frac{1}{d}\log d\left(\log d-\log\log d\right), \end{array}$$

and since $\frac{\log d}{1+\log d}\to 1$ when d→∞, we arrive at the asymptotic lower bound

(20) $$\begin{array}{ccc} C^{NL}\left(\beta ,d\right) & \geq & \frac{1}{d}\log d+o\left(\frac{1}{d}\log d\right). \end{array}$$

To get an upper bound on $C^{NL}\left(\beta ,d\right)$, we write

(21) $$\begin{array}{ccc} C^{NL}\left(\beta ,d\right) & = & \max_{\alpha \in [0,1]}\frac{1}{1+d\alpha }\left(\alpha \log\frac{1}{\alpha }+\left(1-\alpha \right)\log\frac{1}{1-\alpha }\right) \end{array}$$

(22) $$\begin{array}{cc} = & \max_{\alpha \in [0,1]}\frac{\alpha }{1+d\alpha }\log\frac{1}{\alpha }+\frac{1-\alpha }{1+d\alpha }\log\frac{1}{1-\alpha }. \end{array}$$

Since $\left(1-\alpha \right)\log\frac{1}{1-\alpha }\leq \alpha$, we have

(23) $$\begin{array}{c} \frac{1-\alpha }{1+d\alpha }\cdot \log\frac{1}{1-\alpha }\leq \frac{\alpha }{1+d\alpha }\leq \frac{1}{d}, \end{array}$$

so we can continue (22) to obtain

(24) $$\begin{array}{ccc} C^{NL}\left(\beta ,d\right) & \leq & \max_{\alpha \in [0,1]}\frac{\alpha }{1+d\alpha }\cdot \log\frac{1}{\alpha }+\frac{1}{d}. \end{array}$$

Let $\alpha^{*}$ be the value of α that achieves the maximum in (24). The derivative with respect to α of the expression to be maximized on the right-hand side of (24) is

(25) $$\begin{array}{c} f\left(\alpha \right)=-\frac{\log\alpha +d\alpha +1}{{\left(1+d\alpha \right)}^{2}}. \end{array}$$

Since *f* is monotonically decreasing (which can be verified by computing its derivative), positive when α↓0, and negative at α=1, it must have a unique root, therefore $\alpha^{*}$ must satisfy

(26) $$\begin{array}{c} f(\alpha^{*})=0. \end{array}$$

Since

(27) $$\begin{array}{c} f\left(\frac{1}{d}\right)=\frac{1}{4}\left(\log d-2\right) \end{array}$$

is positive for all $d>e^{2}$, we have

(28) $$\begin{array}{c} \alpha^{*}\geq \frac{1}{d} \end{array}$$

for all $d>e^{2}$. Therefore, for large enough *d*,

(29) $$\begin{array}{ccc} \frac{\alpha^{*}}{1+d\alpha^{*}}\log\frac{1}{\alpha^{*}}+\frac{1}{d} & \leq & \frac{1}{d}\log\frac{1}{\alpha^{*}}+\frac{1}{d} \end{array}$$

(30) $$\begin{array}{cc} \leq & \frac{1}{d}\log d+\frac{1}{d} \end{array}$$

(31) $$\begin{array}{cc} = & \frac{1}{d}\log d+o\left(\frac{1}{d}\log d\right), \end{array}$$

where the second inequality follows by (28). Hence, by (24), (31), and the fact that $\alpha^{*}$ achieves the maximum in (24), we get

(32) $$\begin{array}{c} C^{NL}\left(\beta ,d\right)\leq \frac{1}{d}\log d+o\left(\frac{1}{d}\log d\right). \end{array}$$

Combining (20) and (32) proves

(33) $$C^{NL}\left(\beta ,d\right)=\frac{1}{d}\log d+o\left(\frac{1}{d}\log d\right),$$

which, in the asymptotic regime of interest, is equivalent to (15). □

**Remark** **1.** *The converse part of Proposition 3 can also be proven by noting the following. The dead time effectively imposes a constraint on the number of 1s in the output sequence: the proportion of 1s cannot exceed $\frac{1}{d}$. Hence the capacity of this channel can be upper-bounded by the noiseless binary channel without dead time, but with an average-power constraint $\frac{1}{d}$.*

**Figure 2:**
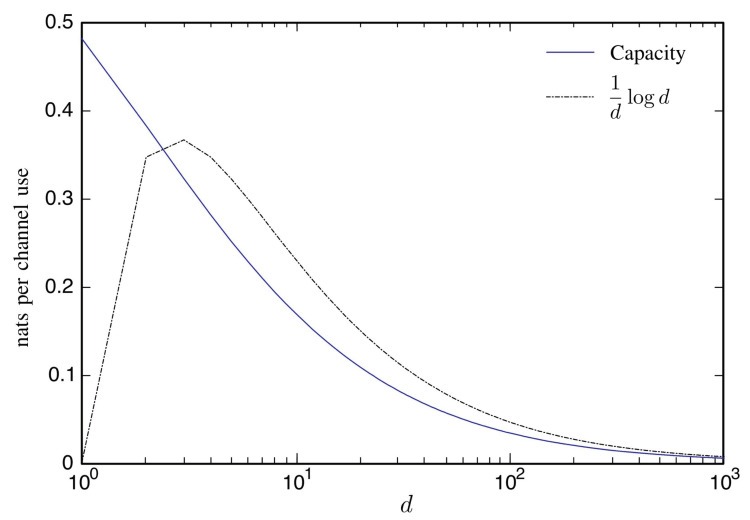
The capacity (7) as a function of *d* compared with the approximation given by (33).

As noted in the proof, when η≥1, the power constraint is inactive, so (33) provides an approximation to (7) when *d* is large. We plot the capacity (7) and its approximation $d\log\frac{1}{d}$ in Figure 2.

### 2.2. Low-Continuous-Time-Power Regime

In this regime, the effect of a fixed dead time affects capacity starting only on the third-order term:

**Proposition** **4.** *In the regime where β↓0 and d is fixed and finite,*

(34) $$\begin{array}{c} C^{NL}\left(\beta ,d\right)=\beta \log\frac{1}{\beta }+\beta -\left(d+\frac{1}{2}\right)\beta^{2}+o\left(\beta^{2}\right). \end{array}$$

**Proof.**  Following the argument used in the proof of Proposition 2, we have that, for small enough β, the maximum in (9) is achieved by

(35) $$\begin{array}{c} \alpha =\frac{\beta }{1-d\beta }. \end{array}$$

Therefore,

(36) $$\begin{array}{ccc} C^{NL}\left(\beta ,d\right) & = & \left(1-d\beta \right)H_{b}\left(\frac{\beta }{1-d\beta }\right). \end{array}$$

Using the Taylor series expansion for small *a*

(37) $$\begin{array}{c} -\left(1-a\right)\log\left(1-a\right)=a-\frac{1}{2}a^{2}+o\left(a^{2}\right), \end{array}$$

we continue (36) as

(38) $$\begin{array}{ccc} C^{NL}\left(\beta ,d\right) & = & \left(1-d\beta \right)\left(\frac{\beta }{1-d\beta }\log\frac{1-d\beta }{\beta }+\frac{1-d\beta -\beta }{1-d\beta }\log\frac{1-d\beta }{1-d\beta -\beta }\right) \end{array}$$

(39) $$\begin{array}{cc} = & \beta \log\frac{1}{\beta }+\left(1-d\beta \right)\log\left(1-d\beta \right)-\left(1-\left(d+1\right)\beta \right)\log\left(1-(d+1)\beta \right) \end{array}$$

(40) $$\begin{array}{cc} = & \beta \log\frac{1}{\beta }-d\beta +\frac{1}{2}d^{2}\beta^{2}+\left(d+1\right)\beta -\frac{1}{2}{\left(d+1\right)}^{2}\beta^{2}+o\left(\beta^{2}\right) \end{array}$$

(41) $$\begin{array}{cc} = & \beta \log\frac{1}{\beta }+\beta -\left(d+\frac{1}{2}\right)\beta^{2}+o\left(\beta^{2}\right), \end{array}$$

which is as claimed. □

## 3. The Poisson Channel

To introduce our model of the discrete-time Poisson channel with dead time, we start with a continuous-time picture. As in the noiseless case, we assume that the receiver employs a single-photon detector with a time resolution of *t* seconds: the time axis is divided into *t*-second slots, and each detected photon is declared to be in a certain slot. Further assume that a dead-time period lasts τ seconds, where τ=dt for an integer *d*. The transmitter modulates a laser signal by a (properly normalized) nonnegative waveform w(·). Let *Y* be the number of detected photons within a slot $\left[t_{0},t_{0}+t\right)$. Assume there is no dark current. Then, provided that the channel is not dead at time $t_{0}$,

(42) $$\mathrm{Pr}\left(Y=0\right)=1-\mathrm{Pr}\left(Y=1\right)=e^{-x},$$

where

(43) $$x=\int_{t_{0}}^{t_{0}+t}w\left(s\right)\;ds.$$

Note that (42) is different from the channel law of a standard Poisson channel, for which *Y* can take values that are larger than 1. This is because we assume the dead time τ to be longer than one slot *t*, and consequently there cannot be more than one recorded photon within one slot.

Thus, formally, the discrete-time Poisson channel with nonparalyzable dead time *d* has input alphabet $R_{0}^{+}$, output alphabet {0,1}, and channel law

(44) $$\begin{array}{c} \mathrm{Pr}\left(Y_{i}=1|X^{i}=x^{i},Y^{i-1}=y^{i-1}\right)=\left\{\begin{array}{cc} 1-e^{-x_{i}}, & \mathrm{if}\;y_{j}=0\;\mathrm{for}\;\mathrm{all}\;j\in \left\{i-d,\ldots ,i-1\right\}\\ 0, & \mathrm{otherwise}. \end{array}\right. \end{array}$$

To model paralyzable dead time, we introduce an auxiliary sequence ${\hat{Y}}^{n}$, which describes the output of the channel (42) without dead time:

(45a) $$\begin{array}{c} \mathrm{Pr}\left({\hat{Y}}_{i}=1|X^{i}=x^{i},{\hat{Y}}^{i-1}={\hat{y}}^{i-1}\right)=1-e^{-x_{i}}. \end{array}$$

We then model the output sequence $Y^{n}$ as follows: $X^{n}$ 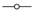 ${\hat{Y}}^{n}$ 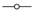 $Y^{n}$ form a Markov chain, and

(45b) $$\mathrm{Pr}\left(Y_{i}=1|{\hat{Y}}^{i}={\hat{y}}^{i},Y^{i-1}=y^{i-1}\right)=\left\{\begin{array}{cc} 1, & \mathrm{if}\;{\hat{y}}_{i}=1\;\mathrm{and}\;{\hat{y}}_{j}=0\;\mathrm{for}\;\mathrm{all}\;j\in \left\{i-d,\ldots ,i-1\right\}\\ 0, & \mathrm{otherwise}. \end{array}\right.$$

Assume that an average-power constraint ρ is imposed on the continuous-time input waveform w(·). By (43), this implies an average-power constraint β=ρt on the discrete-time input *x*. That is, averaged over the codebook and the blocklength,

(46) E[X]≤β.

**Remark** **2.** *The capacities of the two channels (44) and (45a,b) under the constraint (46) are in general not equal. For simplicity of exposition, in the rest of this section we shall focus on nonparalyzable dead time (44). However, all results in this section, namely, Propositions 5–8, hold for paralyzable dead time (45a,b) as well. Indeed, the proofs and derivations in this section apply almost without change to paralyzable deadtime; we shall provide additional explanations when needed.*

We next study the capacity of the Poisson channel with nonparalyzable dead time (44) in the regime where β is close to zero. As in the previous section, we distinguish between the wideband scenario and the low-continuous-time-power scenario. In the former, t↓0, so β↓0 and d→∞ with η=dβ=τρ remaining unchanged; in the latter, ρ↓0 so β↓0 while *d* remains unchanged. In the former, wideband scenario, we further distinguish between the cases where there is feedback and where there is not. Henceforth we shall stay in the discrete-time picture and no longer refer to the continuous-time picture.

For comparison, we note that, for a discrete-time Poisson channel without dead time and with average-power constraint β, in the regime where β is close to zero, the best known capacity approximation is given by [10]

(47) $$\beta \log\frac{1}{\beta }-\beta \log\log\frac{1}{\beta }+O\left(\beta \right).$$

(The work [10] considers the standard Poisson channel where the output is not binary. However, the achievability proof in [10] maps the output to a binary random variable, so (47) applies also to the binary-output channel that is the same as our model when d=0.) In general, no closed-form capacity expression has been found for this channel. Several capacity bounds and asymptotic results have been obtained in [7,8,9,10,11].

### 3.1. Wideband Regime, with Feedback

Consider the regime where β↓0 while η=dβ is held fixed. Assume that immediate noiseless feedback is available, so the encoder learns the realizations of $Y_{1},\ldots ,Y_{i-1}$ before producing $x_{i}$. We first observe that the capacity of the Poisson channel with feedback cannot exceed that of the noiseless binary channel.

**Proposition** **5.** *For any $\beta \in R_{0}^{+}$ and $d\in Z_{0}^{+}$, the capacity of the channel (44) under constraint (46) with immediate noiseless feedback satisfies*

(48) $$C_{\mathrm{FB}}^{P}\left(\beta ,d\right)\leq C^{NL}\left(\beta ,d\right).$$

**Proof.**  Assume that an encoder-decoder pair for the Poisson channel with feedback is given. When the encoder is applied on the message M=m, the output vector equals $y^{n}$ with a certain probability

(49) $$\mathrm{Pr}(Y^{n}=y^{n}|M=m).$$

Now for the noiseless channel we construct a random encoder as follows: given the message M=m, the codeword is chosen to be $y^{n}$ with the probability given by (49). Clearly, all codewords will pass through the channel without change. Combining this random encoder with the given decoder for the Poisson channel, we then obtain exactly the same error probability for both channels. Further, from (44) it is clear that the average power in $Y^{n}$ is less than or equal to that in $X^{n}$, therefore our random encoder for the noiseless channel consumes at most as much power as the given encoder for the Poisson channel. Thus, given any code for the Poisson channel with feedback, we can construct a valid code for the noiseless binary channel that has the same performance. □

The next proposition shows that, in the wideband regime, the capacity of the Poisson channel with feedback has the same dominant term as that of the noiseless binary channel.

**Proposition** **6.** *For the Poisson channel (44) under constraint (46) with immediate noiseless feedback, in the regime where β↓0 and η=dβ is fixed, capacity has the asymptotic expression*

(50) $$C_{\mathrm{FB}}^{P}\left(\beta ,\frac{\eta }{\beta }\right)=\left\{\begin{array}{cc} \beta \log\frac{1}{\beta }+o\left(\beta \log\frac{1}{\beta }\right), & \eta <1\;\\ \frac{\beta }{\eta }\log\frac{1}{\beta }+o\left(\beta \log\frac{1}{\beta }\right), & \eta \geq 1. \end{array}\right.$$

**Proof.**  The converse part follows immediately from Propositions 2, 3 and 5.

We next prove the achievability part in the case where η<1. We shall describe a random coding scheme. We first note a small technicality: the average input power of the following scheme is larger than β and of the form β+o(β). Hence, to obtain a codebook that satisfies the average-power constraint, one should replace β in the following by (1−ϵ)β for some positive ϵ, and later let ϵ approach zero. For simplicity, we shall ignore this technicality henceforth.

Assume that β is sufficiently small. For the first channel use, we choose

(51) $$X=\left\{\begin{array}{cc} -\frac{1}{(1-\eta )\log\beta } & \mathrm{with}\;\mathrm{probability}\;\beta \log\frac{1}{\beta },\\ 0 & \mathrm{otherwise}. \end{array}\right.$$

For future channel uses, based on the feedback, the transmitter determines whether the channel is dead or not. When it is dead, the transmitter chooses X=0 with probability one; otherwise it picks *X* according to (51) independently of all past inputs and outputs.

From (51) we obtain

(52) $$E[X|\mathrm{channel}\;\mathrm{is}\;\mathrm{not}\;\mathrm{dead}]=\frac{\beta }{1-\eta },$$

and

(53) $$\mathrm{Pr}\left(Y=1|\mathrm{channel}\;\mathrm{is}\;\mathrm{not}\;\mathrm{dead}\right)=\beta \log\frac{1}{\beta }\cdot \left(1-e^{\frac{1}{(1-\eta )\log\beta }}\right)=\frac{\beta }{1-\eta }+o\left(\beta \right).$$

Since each Y=1 results in *d* dead channel uses, recalling dβ=η, we have by (53) that, as n→∞ and β↓0, the ratio of dead to not-dead channel uses approaches $\frac{\eta }{1-\eta }$ in probability, and hence the proportion of not-dead channel uses among all channel uses approaches (1−η) in probability. This combined with (52) shows that, as claimed earlier, over all channel uses E[X]=β+o(β).

The dead channel uses can be identified by both the transmitter and the receiver, hence they can be discarded. The remaining channel uses form a sequence that is memoryless, and our choice of inputs on these channel uses are independent and identically distributed (IID). We can hence apply Shannon’s classic result for discrete memoryless channels [17]: over the channel uses that are not dead, we can achieve all rates up to the mutual information. Note that, for small *a*,

(54) $$H_{b}\left(a\right)=a\log\frac{1}{a}+o\left(a\log\frac{1}{a}\right).$$

Recalling (53), we then have

(55) $$H\left(Y|\;\mathrm{channel}\;\mathrm{is}\;\mathrm{not}\;\mathrm{dead}\right)=\frac{\beta }{1-\eta }\log\frac{1}{\beta }+o\left(\beta \log\frac{1}{\beta }\right).$$

From (51) we have

(56) $$H\left(Y|X,\mathrm{channel}\;\mathrm{is}\;\mathrm{not}\;\mathrm{dead}\right)=\beta \log\frac{1}{\beta }H_{b}\left(e^{\frac{1}{(1-\eta )\log\beta }}\right)=o\left(\beta \log\frac{1}{\beta }\right).$$

Hence we have

(57) $$I\left(X;Y|\;\mathrm{channel}\;\mathrm{is}\;\mathrm{not}\;\mathrm{dead}\right)=\frac{\beta }{1-\eta }\log\frac{1}{\beta }+o\left(\beta \log\frac{1}{\beta }\right).$$

Recalling that the proportion of not-dead channel uses tends to (1−η) in probability as n→∞ and β↓0 completes the proof for the case where η<1.

We now consider the case where η≥1. We use a similar random coding scheme as above, the difference being we replace the distribution (51) by the following:

(58) $$X=\left\{\begin{array}{cc} -\frac{1}{\gamma \log\beta } & \mathrm{with}\;\mathrm{probability}\;\beta \log\frac{1}{\beta },\\ 0 & \mathrm{otherwise}, \end{array}\right.$$

where γ>0 will be chosen to approach zero later on. Instead of (53), we now have

(59) $$\mathrm{Pr}\left(Y=1|\mathrm{channel}\;\mathrm{is}\;\mathrm{not}\;\mathrm{dead}\right)=\beta \log\frac{1}{\beta }\cdot \left(1-e^{\frac{1}{\gamma \log\beta }}\right)=\frac{\beta }{\gamma }+o\left(\beta \right).$$

This implies that, as n→∞ and β↓0, the portion of not-dead channel uses approaches $\frac{\gamma }{\eta +\gamma }$ in probability. This further implies that

(60) $$E[X]=E[X]|\mathrm{channel}\;\mathrm{is}\;\mathrm{not}\;\mathrm{dead}\cdot \left(\frac{\gamma }{\eta +\gamma }+o\left(1\right)\right)=\frac{\beta }{\gamma }\cdot \left(\frac{\gamma }{\eta +\gamma }+o\left(1\right)\right)=\frac{\beta }{\eta +\gamma }+o\left(\beta \right),$$

so the average-power constraint is satisfied when β is small enough.

By the same argument as in the previous case, over the channel uses that are not dead, we can achieve any rate up to

(61) $$I\left(X;Y|\;\mathrm{channel}\;\mathrm{is}\;\mathrm{not}\;\mathrm{dead}\right)=\frac{\beta }{\gamma }\log\frac{1}{\beta }+o\left(\beta \log\frac{1}{\beta }\right).$$

The overall rate is thus given by the above multiplied by $\frac{\gamma }{\eta +\gamma }$, which is

(62) $$\frac{\beta }{\eta +\gamma }\log\frac{1}{\beta }+o\left(\beta \log\frac{1}{\beta }\right).$$

Letting γ approach zero completes the proof. □

**Remark** **3.** *The coding schemes used in the above proof work for paralyzable dead time as well. When dead time is paralyzable, then in general whether the channel is dead or not cannot be determined from the output sequence. Nevertheless, for the proposed schemes, it can be so determined. Indeed, since the initial state of the channel is known (“not dead”), and since the transmitter always sends 0 when it knows the channel to be dead, one can show by induction that no arrival will occur when the channel is dead, hence dead time will never be restarted in the paralyzable case. This implies ${\hat{Y}}^{n}=Y^{n}$ with probability one.*

### 3.2. Wideband Regime, without Feedback

Again consider the regime where β↓0 while η=dβ is held fixed. Now we assume there is no feedback, so the transmitter cannot know exactly when the channel is dead. In the following we analyze a strategy in which the transmitter sends zero whenever the channel may be dead. The strategy employs pulse-position modulation (PPM) [10,18,19].

We fix a positive integer *b* and specify its value later. We divide the *n* channel uses into blocks of length (b+d). Within each block, we send a PPM symbol in the first *b* channel uses, and send zeros in the last *d* channel uses. Specifically, the transmitter uniformly picks one among the first *b* channel uses, and sends

(63) X=ξ≜β(b+d).

In all other channel uses in this block, it sends X=0. The pulse position in different blocks are chosen independently. Clearly, the power constraint (46) is satisfied.

Within a specific block, given the input vector, the output has the following distribution. With probability $1-e^{-\xi }$, Y=1 at the same position as the input pulse, and Y=0 elsewhere; and with probability $e^{-\xi }$, Y=0 for the entire block. Furthermore, the channel’s behavior between different blocks are independent (because the last *d* channel uses in every block are not used, a dead-time period cannot extend to the next block). We thus obtain $\left\lfloor \frac{n}{b+d}\right\rfloor$ uses of a memoryless *b*-ary erasure channel, where the erasure probability is $e^{-\xi }$, and with IID uniform inputs. The capacity of this erasure channel is given by

(64) $$(1-e^{-\xi })\cdot \log b.$$

The rate we thus achieve over the Poisson channel is (ignoring the difference between $\frac{n}{b+d}$ and $\left\lfloor \frac{n}{b+d}\right\rfloor$, whose effect vanishes for large *n*)

(65) $$\frac{1-e^{-\xi }}{b+d}\log b.$$

We now choose

(66) b=⌊ϵd⌋

for some positive ϵ, and recall that η=bβ. Then (65) becomes

(67) $$\frac{1-e^{-(1+\epsilon )\eta }}{(1+\epsilon )\eta }\cdot \beta \log\frac{\eta }{\beta }.$$

(In the above we ignored the difference between ⌊ϵd⌋ and ϵd, the effect of which becomes negligible as d→∞.) Letting ϵ↓0 in the above yields the following asymptotic lower bound.

**Proposition** **7.** *For the channel (44) under constraint (46) with no feedback, in the regime where β↓0 and η=dβ is fixed, capacity satisfies*

(68) $$C^{P}\left(\beta ,\frac{\eta }{\beta }\right)\geq \frac{1-e^{-\eta }}{\eta }\cdot \beta \log\frac{1}{\beta }+o\left(\beta \log\frac{1}{\beta }\right),\;\eta >0.$$

In the above scheme, the transmitter sends 0 whenever there is a nonzero probability that the channel is dead, hence no input energy is wasted. The penalty of this approach is that most of the channel uses are wasted because they are treated as “possibly dead.” Each “possibly dead” period spans *d* channel uses, and we must use all the energy budget that is “saved” within this period, which equals η, before the next “possibly dead” period starts. This means our choice of the amplitude of the pulse in PPM must be at least η. For the Poisson channel without dead time, the asymptotically optimal choice for the amplitude of the pulse in PPM should approach zero [10]. That we must choose η in place of 0 accounts for the factor $\frac{1-e^{-\eta }}{\eta }$ in (68) as opposed to 1 in (47). One can check that using on-off keying instead of PPM (while still sending zero whenever the channel may be dead) achieves the same lower bound.

An alternative approach to the above would be to “waste” energy instead of channel uses, or to find a trade-off between these two resources. At the time of writing this paper, we have not found a scheme along this direction that provides a better asymptotic lower bound than (68). Neither have we found a nontrivial upper bound; the best asymptotic upper bound that we know is the capacity with feedback (50).

**Figure 3:**
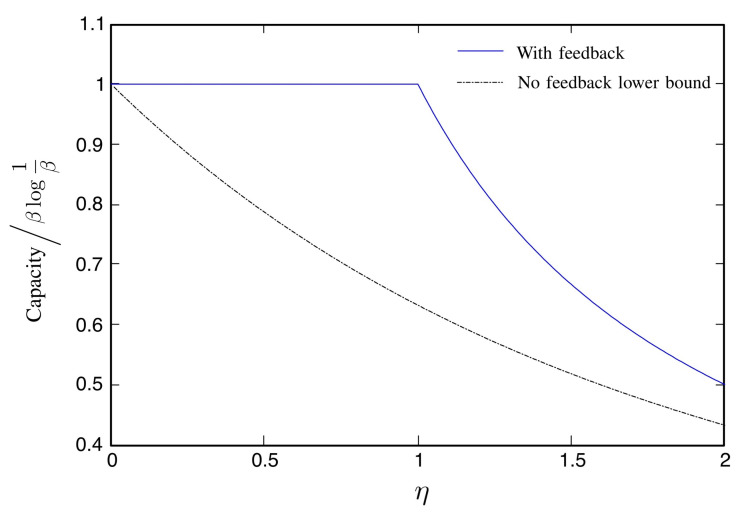
The Poisson channel in the wideband regime: comparison between the asymptotic capacity in the presence of feedback (50) and the lower bound when there is no feedback (68). Both expressions are divided by $\beta \log\frac{1}{\beta }$ and taken to the limit where β↓0, i.e., we compare the scaling constant in front of the dominant term $\beta \log\frac{1}{\beta }$.

We compare the asymptotic capacity with feedback (50) and the lower bound without feedback (68) in Figure 3.

### 3.3. Low-Continuous-Time-Power Regime

We now consider the regime where the allowed continuous-time input power is low, i.e., where β↓0 while *d* is held fixed. We shall not consider feedback, because, as we shall see, the effect of dead time on capacity is rather small even when there is no feedback.

We again consider a PPM scheme. The *n* channel uses are divided into blocks of length (b+d), where we now choose

(69) $$b=\left\lfloor \frac{1}{\beta \log\frac{1}{\beta }}\right\rfloor .$$

Within each block, the transmitter uniformly picks one among the first *b* channel uses, where it sends

(70) $$X=\zeta ≜\frac{1}{\log\frac{1}{\beta }}.$$

For all the other channel uses in this block, the transmitter sends 0. The pulse position in different blocks are chosen independently of each other. The average-power constraint (46) is satisfied: over all the *n* channel uses,

(71) $$E[X]\leq \frac{\zeta }{b+d}\leq \beta .$$

As in Section 3.2, we obtain $\left\lfloor \frac{n}{b+d}\right\rfloor$ uses of a *b*-ary erasure channel. For each block, with probability $e^{-\zeta }$, the output sequence contains only zeros; and with probability $\left(1-e^{-\zeta }\right)$, Y=1 at the same position as the input pulse, and all other outputs in the block are zero. The capacity of this *b*-ary erasure channel is

(72) $$(1-e^{-\zeta })\log b.$$

The rate we can thus achieve over the original channel is (ignoring the effect of the ⌊·⌋ operation)

(73) $$\frac{(1-e^{-\zeta })\log b}{b+d}=\frac{\left(1-e^{-\frac{1}{\log\frac{1}{\beta }}}\right)\log\left\lfloor \frac{1}{\beta \log\frac{1}{\beta }}\right\rfloor }{\left\lfloor \frac{1}{\beta \log\frac{1}{\beta }}\right\rfloor +d}=\beta \log\frac{1}{\beta }-\beta \log\log\frac{1}{\beta }+O\left(\beta \right).$$

Comparing this expression with (47) shows that the influence of *d* is only in the O(β) term. We summarize this observation in the following proposition.

**Proposition** **8.** *For the channel (44) under constraint (46), in the regime where β↓0 and d is fixed, capacity satisfies*

(74) $$C^{P}\left(\beta ,d\right)=\beta \log\frac{1}{\beta }-\beta \log\log\frac{1}{\beta }+O\left(\beta \right),$$

*for all finite d.*

## 4. Discussion

As a first step toward understanding communication channels with detector dead time, we have studied the noiseless binary channel and the discrete-time Poisson channel with dead time in the asymptotic regime where the allowed average input power approaches zero. Although these channels have memory, the results in this paper were obtained using simple tools; we have not explored information stability [20] or information-spectrum methods [21,22].

In the scenario where bandwidth is fixed and the average continuous-time input power is required to be low, for both channels, dead time has no effect on the first- and second-order terms in capacity. This may not be surprising, as only a vanishing proportion of channel uses are dead. In the scenario where continuous-time input power is fixed and bandwidth grows large, if dead time limits the maximum possible output rate (as compared to the allowed input rate), then it incurs a penalty in the dominant term in capacity for both channels. If dead time does not limit the output rate, then, for the noiseless channel and for the Poisson channel with feedback, it does not affect the dominant term in capacity, even though now a nonvanishing proportion of channel uses would be dead. For the Poisson channel without feedback in this scenario, there is a gap in the dominant term between our best achievability result and the capacity without dead time. Intuitively, this gap is due to the fact that the transmitter must either sacrifice almost all available channel uses (if it sends nothing when the channel “may be dead”), or risk wasting input energy. We conjecture that the dominant term in capacity in this case is indeed smaller than that without dead time, but a nontrivial upper bound remains to be proven.

In our study of the Poisson channel, we have assumed the dark current to be zero. In the presence of a nonzero dark current, detections can occur at the receiver even when no input is provided to the channel, and these detections will trigger the dead time, complicating the problem considerably. We leave this as a topic for future work.

Another potentially interesting problem is the peak-limited continuous-time Poisson channel [4,5,6]. The closed-form capacity expression of this channel is a classic result. However, we are not aware of any capacity results on such a channel with dead time.

## Footnotes

1 Number states (Fock states) are pure quantum states that contain deterministic numbers of photons. They are different from coherent states, which are used to model light emitted by lasers. A (nonzero) coherent state is also a pure quantum state, but contains a random number of photons following a Poisson distribution. For more details we refer the reader to [16].

2 For physicists, the sender sending a photon at a specific time really means that it sends the number state |1〉 in an optical mode centered at that time. Here we assume that the available optical bandwidth is sufficiently large so that each *t*-second slot accommodates a large number of optical modes, and that the time uncertainty of each mode can be ignored. Note that in the text we use “bandwidth” to refer to $\frac{1}{t}$—the reciprocal of the detector’s time resolution—and not the optical bandwidth.

## Abbreviations

The following abbreviations are used in this manuscript:

IID	independent and identically distributed
PPM	pulse-position modulation
RLL	run-length limited

## Appendix A

In this appendix we prove Proposition 1. We first derive capacity under a stronger constraint which requires

(A1) $$\sum_{i=1}^{n}x_{i}\leq \left\lfloor n\beta \right\rfloor \;\mathrm{for}\;\mathrm{every}\;\mathrm{codeword}x^{n}.$$

We then show that this capacity equals $C^{NL}\left(\beta ,d\right)$.

Let N(n,d,q) denote the number of length-*n* sequences containing *q* 1s, and satisfying that each 1 (except the last) must be followed by at least *d* 0s. The total number of possible distinct output sequences, for both channels (4) and (5) under (A1), is then given by

(A2) $$\sum_{q=1}^{\left\lfloor n\beta \right\rfloor }N\left(n,d,q\right).$$

It follows that the capacities of the channels (4) and (5) under constraint (A1) are equal and given by

(A3) $$C^{\prime }\left(\beta ,d\right)=\lim_{n\to \infty }\frac{\log\sum_{q=1}^{\left\lfloor n\beta \right\rfloor }N\left(n,d,q\right)}{n}.$$

To compute N(n,d,q), note that the concerned sequences can be generated by distributing *q* 1s among n−(q−1)d positions, and then adding *d* 0s after each 1 except the last. Therefore,

(A4) N(n,d,q)=n−(q−1)dq,q≤n+d1+d0,otherwise.

We upper-bound the summation in (A3) by upper-bounding each summand by the largest summand, and bounding the number of summands by *n*. This gives

(A5) $$\begin{array}{c} \sum_{q=1}^{\left\lfloor n\beta \right\rfloor }N\left(n,d,q\right)\leq n\max_{q\leq \lfloor n\beta \rfloor }N\left(n,d,q\right). \end{array}$$

On the other hand, clearly

(A6) $$\begin{array}{c} \sum_{q=1}^{\left\lfloor n\beta \right\rfloor }N\left(n,d,q\right)\geq \max_{q\leq \lfloor n\beta \rfloor }N\left(n,d,q\right). \end{array}$$

Combining (A3), (A5) and (A6), and noting that $\frac{\log n}{n}\to 0$ as n→∞, we obtain

(A7) $$\begin{array}{c} C^{\prime }\left(\beta ,d\right)=\lim_{n\to \infty }\frac{\log\max_{q\leq \lfloor n\beta \rfloor }N\left(n,d,q\right)}{n}. \end{array}$$

For $q\leq \frac{n+d}{1+d}$, define

(A8) $$\begin{array}{c} \alpha ≜\frac{q}{n-(q-1)d}. \end{array}$$

By (A4) and Equation (11.40) in [17], we have

(A9) $$nH_{b}\left(\alpha \right)-\log\left(n+1\right)\leq \log N\left(n,d,q\right)\leq nH_{b}\left(\alpha \right).$$

As *n* grows large, α as defined by (A8) can be arbitrarily close to any real number that satisfies

(A10) $$\begin{array}{c} \frac{\alpha }{1+d\alpha }\leq \beta . \end{array}$$

Thus we can combine (A7) and (A9) to obtain

(A11) $$C^{\prime }\left(\beta ,d\right)=\max_{\alpha :\frac{\alpha }{1+d\alpha }\leq \beta }\frac{1}{1+d\alpha }H_{b}\left(\alpha \right).$$

Finally, we show that $C^{NL}\left(\beta ,d\right)=C^{\prime }\left(\beta ,d\right)$ for all β and *d*. Since $C^{\prime }\left(\beta ,d\right)$ is the capacity subject to a stronger constraint than $C^{NL}\left(\beta ,d\right)$, we immediately have $C^{NL}\left(\beta ,d\right)\geq C^{\prime }\left(\beta ,d\right)$. For the reverse direction, consider a sequence of length-*n* codebooks at rate *R* that satisfies the average-power constraint. Take an arbitrary ϵ>0. Form a new sequence of codebooks by collecting all the codewords in the original codebooks that contain at most ⌊(1+ϵ)nβ⌋ 1s. Clearly, the new codebook satisfies the hard constraint for average power (1+ϵ)β. The size of the new codebook must be at least $\frac{\epsilon }{1+\epsilon }\cdot e^{nR}$. As *n* grows large, the influence of the factor $\frac{\epsilon }{1+\epsilon }$ on the rate of the codebook vanishes. We thus obtain, for any ϵ>0,

(A12) $$C^{NL}\left(\beta ,d\right)\leq C^{\prime }\left(\left(1+\epsilon \right)\beta ,d\right).$$

The proof is completed by noting that $C^{\prime }\left(\cdot ,d\right)$ is a continuous function.
